# Impact of NPS MedicineWise general practitioner education programs and Choosing Wisely Australia recommendations on prescribing of proton pump inhibitors in Australia

**DOI:** 10.1186/s12875-020-01158-1

**Published:** 2020-05-09

**Authors:** Jianyun Wu, Scott Dickinson, Zain Elgebaly, Suzanne Blogg, Aine Heaney, Yien Soo, Benjamin Daniels, Lynn Weekes

**Affiliations:** 1 NPS MedicineWise, Level 7, 418A Elizabeth Street, Surry Hills, NSW 2010 Australia; 2 Medicines Policy Research Centre, Centre for Big Data Research in Health, University of New South Wales, Lowy Cancer Research Building, Kensington, NSW 2052 Australia

**Keywords:** Proton pump inhibitors, PPI, Quality use of medicines, General practice, Education, Choosing Wisely Australia, NPS MedicineWise programs

## Abstract

**Background:**

This study evaluated the impact of multifaceted NPS MedicineWise programs that targeted all general practitioners (GPs) in Australia in 2009 and 2015 with the aim of reducing unnecessary prescribing of proton pump inhibitors (PPIs) and encouraged stepping down to a lower strength PPI or to discontinue treatment. The 2015 intervention coincided with the release of Choosing Wisely Australia recommendations from the Royal Australian College of General Practitioners (RACGP).

**Methods:**

Outcome measures included monthly dispensing rates of different strength PPIs prescribed by GPs to concessional patients in Australia. All PPIs were categorized according to the May 2019 revised classifications for standard and low strength PPIs except for esomeprazole 40 mg which was classified as a standard strength and esomeprazole 20 mg as low strength for this analysis. Time series analyses was conducted of the dispensing rates of PPI prescriptions for concessional patients between January 2006 and June 2016 using the Pharmaceutical Benefits Scheme (PBS) and Medicare Benefits Schedule (MBS) databases in Australia. Participants were GPs with dispensed PPI prescriptions to concessional patients between January 2006 and June 2016.

**Results:**

Following the 2009 NPS MedicineWise program we observed a 6.7% reduction in the expected dispensing rate of standard strength PPIs for concessional patients between April 2006 and March 2015, and an 8.6% reduction between April 2009 and June 2016 following the 2015 program launch. We observed a significant increase of 5.6% in the dispensing rate of low strength PPIs for concessional patients between April 2009 and March 2015, and no significant change in trend following the 2015 program.

**Conclusions:**

The NPS MedicineWise programs were associated with reductions in the dispensing rate of standard strength PPIs by June 2016 and an increase in the dispensing rate of low-strength PPIs by March 2015 although this trend did not continue following the 2015 program. This suggests that GPs are stepping down patients to lower strength PPIs following the educational programs. However, lower strength PPIs are still not the majority of PPIs dispensed in Australian and regular interventions to sustain and improve PPI management by GPs may be warranted.

## Background

Proton pump inhibitors (PPIs) are used to treat symptoms of gastro-oesophageal reflux disease (GORD), peptic ulcer disease, functional dyspepsia, Barrett’s oesophagus, and oesophagitis [1–3]. While PPIs are generally well tolerated, their use has been linked to an increased risk of several adverse outcomes [4–9]. Clinical guidelines recommend that patients receive treatment for 4 to 8 weeks on standard strength PPIs and step down to a lower strength or discontinue treatment thereafter. A limited number of indications may require ongoing maintenance treatment [1, 10]. Despite these recommendations, there is growing evidence that PPIs are being inappropriately prescribed in contemporary practice, leading to overuse in both primary care and hospital settings [11–13]. In Australia PPI usage increased from 44.0 defined daily doses (DDDs) per 1000 population in 2000 to 77.5 in 2015 [14], and the highest strengths of each PPI medicine available (Table 1) constitute the majority of dispensed PPI treatment [15].

**Table 1:** PBS item codes used in the analysis of the NPS MedicineWise programs

Drug name	Strength category	mg
**Esomeprazole**	**High strength**^**a**^	**40 (classified as standard for this study)**
	**Standard strength**^**a**^	**20 (classified as low for this study)**
**Lansoprazole**	**Standard strength**	**30**
	**Low strength**	**15**
**Omeprazole**	**Standard strength**	**20**
	**Low strength**	**10**
**Pantoprazole**	**Standard strength**	**40**
	**Low strength**	**20**
**Rabeprazole**	**Standard strength**	**20**
	**Low strength**	**10**

^**a**^ For the purposes of our analyses, we considered the highest available strength esomeprazole (40 mg) as a standard strength PPI, and the lowest available strength esomeprazole (20 mg) as a low strength PPI

Terminology for PPI strength has changed from highest, high and low to high, standard and low since 1 May 2019 [16]. For simplicity we included the only high strength PPI, esomeprazole 40 mg, as a standard strength and esomeprazole 20 mg standard strength as low strength for our analysis (Table 1).

In the past decade, initiatives such as educational programs conducted by NPS MedicineWise and recommendations by Choosing Wisely Australia [1, 17, 18] have been launched with the aim of improving the quality use of PPI medicines and reducing the risk of PPI-related adverse outcomes. The Choosing Wisely program is a physician-led initiative that aims to encourage discussions between physicians and patients around practices that provide little value or may unnecessarily harm patients [17, 19]. In Australia, the program is facilitated by NPS MedicineWise, a national, not-for-profit organisation seeking to improve the quality use of medicines and medical tests in Australia through educational campaigns that target general practitioners (GPs), pharmacists, other health professionals and consumers.

In 2009 and 2015, NPS MedicineWise launched two multifaceted programs targeting GPs with messages about the quality prescribing of PPIs [20, 21]. These programs promoted the appropriate use of step-down PPI treatment approaches.

The 2009 and 2015 NPS MedicineWise PPI programs sought to provide and reinforce evidence-based recommendations to guide GPs in the appropriate primary care management of GORD, and to promote dialogue between GPs and patients about the relative benefits, risks, harms and costs of treatments. The programs included a review of recent safety updates from the Australian Therapeutic Goods Administration and tools for GPs to facilitate step-down PPI therapy in patients whose reflux symptoms were well-controlled [20].

GPs were provided with feedback detailing their prescribing behaviours during the calendar years preceding each intervention (2008 and 2014). This feedback took the form of an individualised report sent to all registered and practicing GPs in Australia. The report used national medicines dispensing records from the Pharmaceutical Benefits Scheme (PBS) to summarise all dispensed PPI medicines prescribed by each GP and compared the data for each medicine to that of all other GPs in Australia. The PBS data consists of administrative dispensing data and contains information that a prescriber writes on a prescription pad that a patient then takes to a pharmacy where the medicine is dispensed to the patient. The report also highlighted how their prescribing aligned with best practice recommendations. This feedback was provided to approximately 20,000 GPs in 2009 and 24,000 GPs in 2015. It included the number of PPI prescriptions dispensed each month, the strengths and the total cost of the prescribed PPIs, and the estimated number of patients receiving long-term PPI treatment for each GP. Long-term PPI treatment was defined as having six or more PPI prescriptions dispensed, which is equivalent to 6 months supply. The feedback does not include the reason for prescribing PPIs and whether it was appropriate or not as this information is not available in the PBS dataset.

In 2009 and 2015, these feedback reports were followed by a clinical audit and a case study for a sample of GPs who had received the reports. See Table 2 for the details and the reach of each of these aspects of the interventions.

**Table 2:** NPS MedicineWise interventions targeting PPI prescribing in Australia

Intervention	Date	Details	Number GPs reached	
			2009	2015
PBS feedback	2009 and 2015	A personalised prescribing report was sent to registered practicing GPs in Australia. The distribution of feedback reports was coordinated with the Department of Human Services by using PBS data.	19,790	24,188
Case study	2009 and 2015	The educational activity explored how to manage a new PPI therapy according to best practice recommendations and it provided GP participants with immediate information on how their prescribing compared with their peers	169	397
Clinical Audit	2009 and 2015	GPs review their current prescribing practice for their patients compared with current best practice guidelines	1590	687

The key recommendations to GPs in the NPS MedicineWise programs in 2009 and 2015 were:

- Review all patients currently being treated with PPIs
- Confirm that the indication for treatment remains, evaluate whether the strength and frequency of PPI dosing can be reduced, and evaluate if PPI therapy can be discontinued
- Encourage lifestyle modifications and review the concomitant use of medicines that may exacerbate symptoms
- Decrease PPI treatment to low strength or intermittent, symptom-driven therapy once symptoms are controlled
- Always discuss the expected duration of treatment and have a plan for stepping down or discontinuing treatment when PPI treatment is initiated.

The aim of this study was to evaluate the impact of these interventions in terms of the changes in monthly dispensing rates of different strength PPIs prescribed by GPs to concessional patients in Australia.

## Methods

### Setting and data

Australia maintains a universal healthcare system entitling all citizens and permanent residents to subsidised medicines through the PBS and subsidised outpatient medical services through the Medicare Benefits Schedule (MBS). The Australian Government Department of Human Services (DHS), now Services Australia, which is the administering body for the PBS and MBS, supplied summary data of monthly dispensing records of PPI medicines subsidised through the PBS, prescribed by a GP, for each GP; and monthly records of every medical service [22] billed to the government by each GP in Australia from January 2006 through to June 2016. GPs included in this study were registered general practitioners, trainees and non-vocationally recognised doctors. The DHS generated a unique identifier code for each GP in the data and this code allowed us to link dispensing and services data. Consent was sought from and provided by the DHS for this study.

PBS data capture prescription medicine dispensing that has resulted in a subsidy paid by the PBS; the data do not capture dispensing of medicines priced below the PBS co-payment threshold or medicines dispensed privately. The price of many PPI medicines is below the general PBS co-payment threshold (range: $29.50 – $38.30 between 2006 and 2016) but above the concessional PBS co-payment threshold (range: $4.70 - $6.20 between 2006 and 2016). To ensure complete ascertainment of PPIs dispensed during the study period, we restricted our analyses to concessional beneficiaries [23].

### Measures

As actual GP prescribing records are not available in our dispensing data, we examined the impact of the NPS MedicineWise interventions on the rate of dispensed PPI prescriptions issued by GPs as a surrogate measure of GP prescribing. Our outcome measures were constructed as the monthly number of standard and low strength PPIs (Table 1) dispensed through the PBS (numerators) per 1000 reimbursable GP consultations (denominator) [24, 25]. In the case of esomeprazole, the only PBS-subsidised strengths available in Australia are classified as “high” and “standard” strength. As discussed previously, for the purpose of our analyses, we considered dispensing’s of the highest available strength esomeprazole (“high”) as a standard strength PPI, and the lowest available strength esomeprazole (“standard”) as a low strength PPI (Table 1).

### Statistical analysis

We used time series intervention models with an autoregressive residual process to analyse the dispensing rate of standard and low strength PPIs separately. We adjusted our data seasonally to account for the well-known “stockpiling” phenomenon that results in increased dispensing of many medicines subsidised through the PBS towards the end of each calendar year, and the subsequent reduced dispensing during the following January and February [23]. We conducted our analyses using the seasonally adjusted data but added isolated seasonal components to the final graphic presentations.

We used two separate change-in-trend variables to represent the NPS MedicineWise interventions in 2009 and 2015. The change-in-trend variable was set as the number count start from 1 since the beginning of the intervention, and all 0 s before the intervention. We hypothesised that the impact of the 2009 intervention would diminish with time and included a decay parameter for the 2009 program in our models to test this hypothesis. If the estimated decay effect was not statistically significant, we set the decay parameter to zero and re-estimated the model. We did not include a decay term for the 2015 intervention as fewer time points were available between April 2015 and the end of our data series (June 2016). We used a linear underlying trend to fit the dispensing rate for standard strength PPIs and a square root of linear trend to represent the underlying trend in the dispensing rate for low strength PPIs. We also examined stationarity and autocorrelation in the residuals after fitting the regression components. If the residuals were auto-correlated, the model also included an autoregressive error term.

The initial model to analyse the dispensing rate of standard and low strength PPIs was:

$$ {y}_t={\beta}_0+{\beta}_1t+{\beta}_2{\mathrm{NPS}}_{09}\exp \left(-\lambda {\mathrm{NPS}}_{09}\right)+{\beta}_3{\mathrm{NPS}}_{15}+{N}_t $$

where *y*_*t*_ represents the seasonally adjusted data in month t, *β*_0_ + *β*_1_*t* represents an underlying linear trend (for the model of the low strength PPIs it is ${\beta}_0+{\beta}_1\sqrt{t}$), NPS_09_ and NPS_15_ are change-in-trend variables representing the 2009 and 2015 intervention programs, respectively, *λ* is a decay parameter and *N*_*t*_ was an autoregressive process with order *p*.

We used the statistical package *mgcv* to perform seasonal adjustment of the data series [26] and estimated the intervention models using generalised non-linear least squares with the package *nlme* [27]. We performed all analyses in R v3.3.3 [28] and used a *p*-value of less than 0.05 to indicate statistical significance.

## Results

We found that the 2009 NPS MedicineWise program was associated with a statistically significant reduction in the dispensing rate of standard strength PPIs (*p* < 0.0001; Table 3) and a significant increase in the rate of low strength PPI dispensing (*p* < 0.0001; Table 3). The 2015 NPS MedicineWise program was associated with a statistically significant reduction in the dispensing rate of standard strength PPIs (*p* < 0.0001) but no change in the rate of low strength PPI dispensing (Table 3).

**Table 3:** Generalised linear and non-linear least square estimates of standard and low strength PPIs monthly time series data between January 2006 and June 2016

Variables in the Model	Standard Strength PPIs			Low Strength PPIs		
	Coefficient	95% CI	*p*-Value	Coefficient	95% CI	*p*-Value
Intercept (*β*_0_)	70.077	(69.09, 70.06)	< 0.0001	13.546	(12.98, 14.17)	< 0.0001
t (*β*_1_)	0.163	(0.13, 0.20)	< 0.0001			
$\sqrt{\mathrm{t}}$ (*β*_1_)				1.563	(1.43, 1.67)	< 0.0001
*NPS*_09_ (*β*_2_)	−0.153	(−0.20, −0.11)	< 0.0001	0.282	(0.22, 0.36)	< 0.0001
Decay (*λ*)	–	–	–	0.047	(0.036, 0.062).	< 0.0001
*NPS*_15_(*β*_3_)	−0.334	(− 0.46, − 0.21)	< 0.0001	0.030	(− 0.028, 0.087)	0.296
Auto-correlation ϕ	− 0.193	(− 0.36, − 0.015)	0.0313	–	–	–

**Fig. 1:**
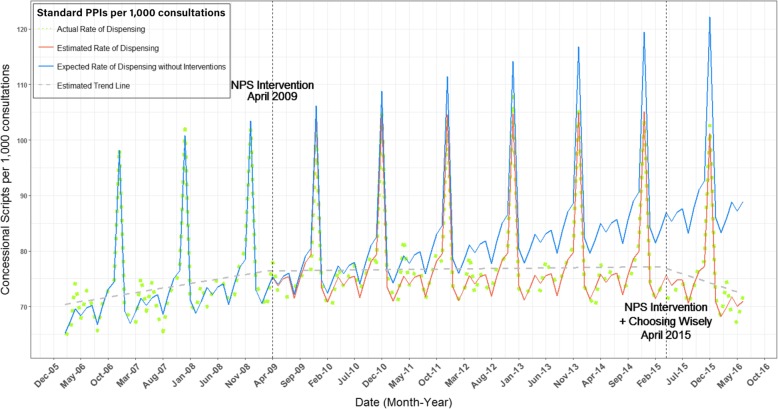
Fitted model for number of concessional standard strength PPIs dispensing per 1000 consultations between January 2006 and June 2016

Compared to the expected rate of standard strength PPI dispensing without the interventions (solid blue line, Fig. 1), we observed a 6.7% reduction in the rate of standard strength PPI dispensing from April 2009 until March 2015, with a total 8.6% reduction by June 2016 since April 2009. The estimated reduction in dispensing of standard strength PPIs was 5.5/1000 GP consultations per month following the 2009 program, and 14.9/1000 GP consultations per month following the 2015 program. We did not observe evidence of a decay effect associated with the 2009 intervention (Table 3).

**Fig. 2:**
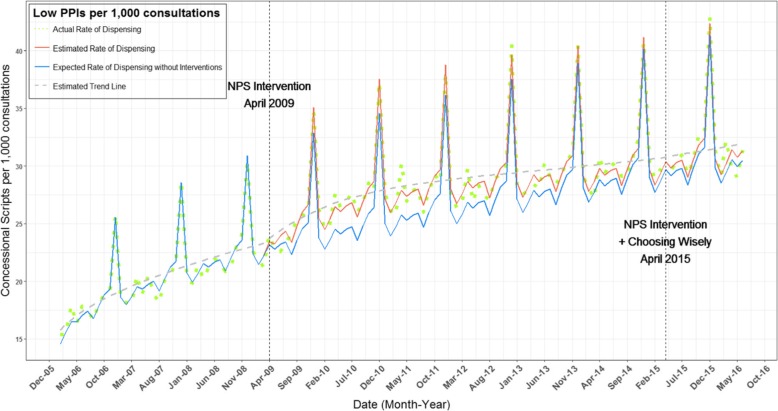
Fitted model for number of concessional low strength PPIs dispensing per 1000 consultations between January 2006 and June 2016

Taking into account the statistically significant decay effect (*p* < 0.0001; Table 3) after the 2009 program, we estimated a 5.6% increase in the dispensing rate of low strength PPIs by March 2015 compared to the expected rate without the intervention (solid blue line; Fig. 2). From April 2009 to June 2016, the overall increase in the dispensing rate of low strength PPIs was about 5.0%. The estimated increase in the dispensing rate of low strength PPIs was 1.5/1000 GP consultations per month following the 2009 program. We observed a slight, but non-significant increase in the rate of low strength PPI dispensing following the 2015 intervention (Table 3).

## Discussion

Our study demonstrated significant changes in the rates of PPI dispensing, as a surrogate measure of GP prescribing, following two targeted interventions, using national, whole-of-population dispensing data. We found that rates of standard strength PPI dispensing declined following the interventions, while dispensing rates for low strength PPIs increased after the 2009 intervention but not the 2015 intervention. These findings suggest that the NPS MedicineWise programs were effective in improving quality PPI prescribing by GPs in Australia.

Our results are consistent with recent studies of Australian veterans, which found that interventions conducted in 2004, 2006 and 2009 by NPS MedicineWise and by the Veterans’ MATES program in 2006 and 2012 resulted in a 20.9% relative decrease in overall PPI dispensing and a 42.2% relative increase in low strength PPI dispensing 12 months after the final intervention in the veteran population [29]. Medicines dispensed to Australian veterans are subsidised through the Repatriation Pharmaceutical Benefits Scheme (RPBS), a funding body distinct from the PBS. Our data did not include RPBS dispensing records and our findings suggest that GPs may have applied new knowledge resulting from these programs in treating both their veteran and other community patients.

We estimated that the 2015 NPS MedicineWise program was associated with a 3.0% decrease in the standard strength PPI dispensing rate in the first 15 months following the intervention. This was greater than the 1.6% decrease during the 15 months after the 2009 intervention and the larger drop may be related to the Choosing Wisely Australia PPI campaign launched during the same month as the NPS MedicineWise 2015 intervention. The Choosing Wisely campaign was aimed at beginning a conversation between GPs and patients around the long-term use of PPIs; with Choosing Wisely members recommending regular attempts at lower strength prescribing or cessation of PPI therapy in patients with uncomplicated disease [18]. Although the impacts of the Choosing Wisely campaign and the 2015 NPS MedicineWise program could not be separated, it is possible that each reinforced the messages of the other, resulting in a further reduction in the observed use of standard strength PPIs.

Other studies have shown the impact of educational programs on GP prescribing, including a study conducted by May et al. Doctors participating in an educational visiting program in Adelaide that focused on better use of prescribed non-steroidal anti-inflammatory drugs (NSAID) reduced their use of NSAIDs by 9 and 28% for two different measures compared to a comparison group [30]. Other studies have found positive impacts of educational programs on GP behaviour, including programs on the treatment and management of incontinence, health behaviours of elderly people and adolescent health care [31–33].

Our study highlights the benefits of engaging with medical practitioners to improve the quality use of medicines. The goal of Choosing Wisely is to start discussions between physicians and patients around specific therapeutic practices. NPS MedicineWise actively engages general practitioners and consumers in educational activities aimed to improve the quality use of medicines through behaviour change [34]. There are potential opportunities for additional quality use of medicines interventions, as well as on-going PPI education.

### Strengths and limitations

Our study evaluated changes to dispensing of PPIs prescribed by GPs to concessional patients over time. The cost of all PPI medicines subsidised by the PBS is above the concessional co-payment threshold and, therefore, we have a complete ascertainment of PPI dispensing for these concessional patients. The DHS began collecting dispensing data for medicines where cost was below the co-payment threshold for general non-concessional patients from April 2012 and, based on dispensing data from that time, it has been estimated that approximately 70% of PPIs prescribed by GPs were dispensed to concessional patients. Nearly 88% of all prescription medicines dispensed under the PBS are for concessional patients. Concessional patients include low-income earners, welfare recipients, and Health Care Card holders and are higher users of health services due to their generally poorer health status. However, we expect the intervention impacts we observed in concessional beneficiaries would be similar among general beneficiaries as we do not expect the treatment of GORD with PPIs to differ between the two subsidy groups.

Most PPIs are available over-the-counter (OTC) in Australia and PPIs that are available from pharmacies without a prescription include rabeprazole, pantoprazole, esomeprazole and omeprazole. Given the limitation of our data, we are not able to determine if patients switched from prescription to OTC PPIs in the study time period. However, during the time period of the study, OTC formulations contained just a 7 day supply and cost was above that of the concessional beneficiary co-payment. We believe it is unlikely that many concessional beneficiaries would have switched to an OTC formulation although we do not know if this is the case. The PBS has a safety net which resets each year on 1 January. In 2019, the PBS Safety Net threshold was $390 for concessional card holders. Before meeting the threshold each medicine costs concessional patients $6.50 and once they reach the threshold all PBS medicines are free of charge. This is an incentive for patients to pay the concessional rates for PBS medicines rather than buying those available OTC from a pharmacist as not only are the medicines cheaper but the cost contributes to the patient reaching the Safety Net threshold. Non-concessional patients also have a safety net but due to the low cost of PPIs they do not have the cost covered under the PBS and are more likely to purchase PPIs OTC than concessional patients. If a doctor will no longer prescribe a PPI to a patient who would still like to take them, they can purchase them OTC. However, the data provided for this study does not include any information on OTC sales.

Although this study is observational and causality cannot be confirmed, given the timing of the intervention programs, and that we did not identify any other potential confounding events following the 2009 and the 2015 programs that might explain the changes we observed, we believe that the change in the rate of PPI dispensing is attributable to the NPS MedicineWise programs and the Choosing Wisely RACGP recommendation.

PBS data are maintained for the purpose of providing reimbursement to patients and pharmacies, and clinical information such as diagnoses and treatment indications are not captured. Although the programs we evaluated were aimed at improving the quality use of PPIs, we are unable to assess the appropriateness of prescribing using these data. Similarly, our prescriber-level PBS data do not allow us to evaluate the rates at which individual patients switched between PPI treatment strengths or ceased PPI therapy or to determine the duration of treatment for individuals.

Another limitation of the study is that there is no information about patient adherence to medicines dispensed.

The strengths of our study is the use of a longitudinal and complete dataset comprised of dispensing records for PPI medicines prescribed to concessional patients by every general practitioner in Australia from 2006 to June 2016. These data allowed for robust estimates of trends over time in the exact population targeted by the interventions.

## Conclusions

The NPS MedicineWise programs were effective in changing the prescribing of PPI medicines by Australian GPs. These programs were complemented by a Choosing Wisely recommendation produced by the RACGP which may have resulted in more efficacious interventions aimed at improving quality prescribing. Regular educational programs targeting the prescribing of PPIs may ensure that quality prescribing practices are continued.

## Data Availability

PBS and MBS data are available online – see the Australian Department of Health - http://www.pbs.gov.au/info/statistics/expenditure-prescriptions/pbs-expenditure-and-prescriptions http://medicarestatistics.humanservices.gov.au/statistics/mbs_item.jsp

## Abbreviations

DDDs: Defined daily doses
GP: General practitioner
MBS: Medicare Benefits Schedule
OTC: Over-the-counter
PBS: Pharmaceutical Benefits Scheme
PPI: Proton pump inhibitor
RACGP: Royal Australian College of General Practitioners
